# Capsaicin Supplementation Reduces Physical Fatigue and Improves Exercise Performance in Mice

**DOI:** 10.3390/nu8100648

**Published:** 2016-10-20

**Authors:** Yi-Ju Hsu, Wen-Ching Huang, Chien-Chao Chiu, Yan-Lin Liu, Wan-Chun Chiu, Chun-Hui Chiu, Yen-Shuo Chiu, Chi-Chang Huang

**Affiliations:** 1 Graduate Institute of Sports Science, National Taiwan Sport University, Taoyuan 33301, Taiwan; 1041302@ntsu.edu.tw (Y.-J.H.); 1010503@ntsu.edu.tw (W.-C.H.); chiu2295@yahoo.com.tw (C.-C.C.); 1040204@ntsu.edu.tw (Y.-L.L.); 2 School of Nutrition and Health Sciences, Taipei Medical University, Taipei 11031, Taiwan; wanchun@tmu.edu.tw; 3 Graduate Institute of Health Industry Technology, Research Center for Industry of Human Ecology and Research Center for Chinese Herbal Medicine, College of Human Ecology, Chang Gung University of Science and Technology, Taoyuan 33303, Taiwan; chchiu@mail.cgust.edu.tw; 4 Department of Orthopedic Surgery, Taipei Medical University Shuang Ho Hospital, New Taipei City 23561, Taiwan

**Keywords:** capsaicin, anti-fatigue, exercise performance, forelimb grip strength, glycogen

## Abstract

Chili pepper is used as a food, seasoning and has been revered for its medicinal and health claims. It is very popular and is the most common spice worldwide. Capsaicin (CAP) is a major pungent and bioactive phytochemical in chili peppers. CAP has been shown to improve mitochondrial biogenesis and adenosine triphosphate (ATP) production. However, there is limited evidence around the effects of CAP on physical fatigue and exercise performance. The purpose of this study was to evaluate the potential beneficial effects of CAP on anti-fatigue and ergogenic functions following physiological challenge. Female Institute of Cancer Research (ICR) mice from four groups (*n* = 8 per group) were orally administered CAP for 4 weeks at 0, 205, 410, and 1025 mg/kg/day, which were respectively designated the vehicle, CAP-1X, CAP-2X, and CAP-5X groups. The anti-fatigue activity and exercise performance was evaluated using forelimb grip strength, exhaustive swimming time, and levels of serum lactate, ammonia, glucose, BUN (blood urea nitrogen) and creatine kinase (CK) after a 15-min swimming exercise. The grip strength and exhaustive swimming time of the CAP-5X group were significantly higher than other groups. CAP supplementation dose-dependently reduced serum lactate, ammonia, BUN and CK levels, and increased glucose concentration after the 15-min swimming test. In addition, CAP also increased hepatic glycogen content, an important energy source for exercise. The possible mechanism was relevant to energy homeostasis and the physiological modulations by CAP supplementation. Therefore, our results suggest that CAP supplementation may have a wide spectrum of bioactivities for promoting health, performance improvement and fatigue amelioration.

## 1. Introduction

Capsaicin (CAP, trans-8-methyl-*N*-vanillyl-6-nonenamide) is, a plant with origins in the Americas. It is a naturally occurring phytochemical and is one of the active ingredients of red and chili peppers [1,2]. Peppers have become a popular culinary spice for food throughout the world. Pepper plants are shallow-rooted and lack a taproot, which are notorious for their sensitivity to moisture stress at flowering and fruit setting. CAP is an odorless white crystal with severe burning pungency [3]. On the other hand, pungency is influenced by the weather conditions such as heat waves and moisture and increases with the growth of the maturity of fruit [4,5]. The gland on the placenta of the fruit produces CAP, mostly located in vesicles or vacuole like sub-cellular organelles of epidermal cells of placenta in the pod [6]. The highest concentrations of CAP are found in the ovary and in the lower flesh (tip) and the lowest content in the plant seeds [7].

Capsaicin has been widely investigated, and is an important molecule in the area of research in medicinal field. Numerous studies have demonstrated that CAP has extensive bioactivities, such as analgesic [8], antioxidant [9], anti-inflammatory, anti-cancer [10], and anti-obesity properties [11,12]. In addition, the CAP is known to increase energy metabolism by proposed mitochondrial function via activation of the TRPV1 (transient receptor potential vanilloid 1) [12]. TRPV1 activation by dietary capsaicin activates mitochondrial respiration, promotes mitochondrial biogenesis, and improves energy metabolism and exercise endurance by upregulating PGC-1α (peroxisome proliferator-activated receptor-γ coactivator-1α) in skeletal muscles [13,14]. TRPV1 channels may also participate in some chronic adaptations induced by regular physical exercise or training programs, including muscular hypertrophy and ATP production in muscle [15].

However, relatively few studies directly address the possible anti-fatigue function of CAP. Fatigue is defined as an exercise-induced inability to perform the expected or desired work output. Physiological fatigue results from excessive physical loading, inadequate rest, or mental strain/pressure and is further classified as central and peripheral fatigue [16]. Physical fatigue can be accompanied by deterioration in functional performance [17]. Exhaustion theory suggests that energy source depletion and excess metabolite accumulation can lead to fatigue [18]. A previous article reviewed the physiological effects on energy metabolism and utilization via TRPV1 activation [15]. Therefore, we conducted this study to evaluate the potential ergogenic and anti-fatigue effects of CAP using our previously established in vivo platform [19,20].

## 2. Materials and Methods

### 2.1. High-Performance Liquid Chromatography (HPLC) Analysis

The CAP used for supplementation in the study was purchased from Nowfoods LLC (Taichung City, Taiwan). The total capsaicin content of CAP product was determined by a high-performance liquid chromatography (HPLC) method. Reversed-phase HPLC was performed using a Hitachi Primaide 1110 HPLC pump system equipped with a Primaide 1430 diode array detector, and a Primaide 1210 autosampler was used to analyze capsaicin on a Phenomenex Luna C18 column (I.D. 4.6 × 250 mm) at 280 nm. A mixture of 5% methanol in 0.1% phosphoric acid was used as the mobile phase at a flow rate of 1 mL/min and an injection volume of 20 μL.

### 2.2. Animals and Experiment Design

Thirty-two 8-week-old female ICR mice in a specific pathogen-free condition were obtained from the BioLASCO (Yi-Lan, Taiwan). The mice were housed in an animal room at a constant temperature (22 ± 1 °C) and humidity (50%–60%) under a 12:12 h light-dark cycle with standard laboratory diet (No. 5001; PMI Nutrition International, Brentwood, MO, USA). The distilled water was provided ad libitum. A total of eight cages housing ICR female mice (*n* = 4 animals per cage) were administrated in the current study. All animal experiments were reviewed and approved by the Animal Care and Use Committee (IACUC) on the ethics of animal experiments at the University of National Taiwan Sport, and this study conformed to guidelines of protocol IACUC-10508 for animal welfare.

After one week of acclimation, the animals were randomly divided into the four groups (*n* = 8 per group in each test) for oral gavage treatment with CAP once a day for 28 consecutive days: (1) vehicle control; (2) 205 mg/kg CAP (CAP-1X); (3) 410 mg/kg CAP (CAP-2X); and (4) 1025 mg/kg CAP (CAP-5X). Vehicle or CAP was administrated by oral gavage. The control group received the vehicle at the same dosage volume of solution equivalent to body weight (BW). The food intake and water consumption were monitored daily, and BW was recorded weekly.

### 2.3. CAP Preparation and Supplementation

CAP extracts was prepared using good manufacturing practices (GMP) and stored at 4 °C for following experiments. The administration dose of CAP was 16.7 mg/kg for human daily recommended intake. The mouse dosage was converted from a human equivalent dose (HED) based on body surface area by the following formula from the US Food and Drug Administration: assuming a human weight of 60 kg, the HED for 1000 (mg)/60 (kg) = 16.67 × 12.3 = a mouse dose of 205 mg/kg; the conversion coefficient 12.3 was used to account for differences in body surface area between mice and humans as we described previously [21].

### 2.4. Sample Collection

All animals were euthanized by 95% CO_2_ after the last treatment, and blood was immediately collected. Blood collected by cardiac puncture was centrifuged at 1500× *g* for 10 min at 4 °C and then sera was collected for −80 °C storage. The liver, skeletal muscle (including gastrocnemius and soleus muscles in the back part of the lower legs), kidney, heart, lung, uterine fat pad (UFP), and brown adipose tissue (BAT) were excised and weighed. The liver and gastrocnemius muscle were collected immediately after saline cleaning. Those samples were maintained at −80 °C until analysis of glycogen content.

### 2.5. Forelimb Grip Strength Test

A low-force testing system (Model-RX-5, Aikoh Engineering, Nagoya, Japan) was used to measure the forelimb grip strength of mice undergoing vehicle or CAP treatments. The amount of tensile force exerted by each mouse was measured using a force transducer equipped with a metal bar (2 mm in diameter and 7.5 cm in length). The mice were allowed to grip the pull bar on the grip wire with only their front paws was steadily pulled back until they lost their grip with the metal bar. The detailed procedures have been described in our previous reports [21,22]. The test of forelimb grip strength was performed after administration of the indicated CAP supplementation for 4 weeks. Grip strength was measured 10 times and the peak tension during each trial was recorded with the attached force gauge. The maximal force (in grams) recorded using this low-force system was used as the grip strength.

### 2.6. Swimming Exercise Performance Test

The swimming exercise performance test was performed as previously described [20]. After 4 weeks of CAP supplementation, a lead sheet (5% equivalent to individual body weight) was attached to the tail of mice for exhaustive swimming challenge. Swimming was performed in plastic containers (65 cm tall and radius 20 cm), filled with water to 40 cm water depth and a temperature of 27 ± 1 °C was maintained. The mice were considered exhausted when they failed to rise to the surface of the water to breathe after 7 s. Swim time to exhaustion was evaluated as the index of exercise performance.

### 2.7. Determination of Fatigue-Associated Biochemical Variables

The effect of CAP supplementation on levels of serum lactate, ammonia, glucose, blood urea nitrogen (BUN), and creatinine kinase (CK) was assessed immediately after exercise. One hour after the last treatment, mice underwent a 15-min swimming test without weight loading. After the swimming exercise, blood samples were immediately collected from the submandibular duct of mice and centrifuged at 1500× *g* and 4 °C for 10 min for serum preparation. The serum was determined by use of an autoanalyzer (Hitachi 7060, Hitachi, Tokyo, Japan) on the same day.

### 2.8. Clinical Biochemical Profiles

At the end of the experimental period, all mice were euthanized by 95% CO_2_ and blood was immediately collected at rest status. Serum was collected by centrifugation and the clinical biochemical variables including AST (aspartate transaminase), ALT (alanine transaminase), albumin, TP (total protein), BUN (blood urea nitrogen), creatinine, CK (creatine kinase), UA (uric acid), total cholesterol (TC), TG (triglycerides) and glucose were measured using an auto analyzer (Hitachi 7060, Hitachi, Tokyo, Japan).

### 2.9. Tissue Glycogen Determination

Since liver and skeletal muscles are the two major tissues for glycogen deposition, we investigated whether glycogen contents of these two target tissues could be elevated by CAP administration. Liver and muscle tissues were excised and stored in −80 °C for glycogen content analysis as we described previously [22]. The weights of related visceral organs were also recorded for body compositions.

### 2.10. Histological Staining of Tissue

Liver, skeletal muscle, heart, kidney and uterine fat pad tissue were removed from the vehicle and experimental groups at the end of experiment, fixed in 10% phosphate-buffered formalin and then embedded in paraffin. Tissues were embedded in paraffin, cut into 4-μm-thick transverse sections, and stained with hematoxylin-eosin for blinded histopathological assessment and examined by light microscopy with a charge-coupled device (CCD) camera (BX-51; Olympus, Tokyo, Japan) by a clinical pathologist.

### 2.11. Statistical Analysis

Data are presented as mean ± standard error of mean (SEM). Statistical differences among groups were analyzed using one-way ANOVA and the Cochran–Armitage test for dose-effect trend analysis with SAS 9.0 (SAS Inst., Cary, NC, USA). The level of statistical significance was set at *p* < 0.05.

## 3. Results

### 3.1. Content of Capsaicin in CAP

**Figure 1:**
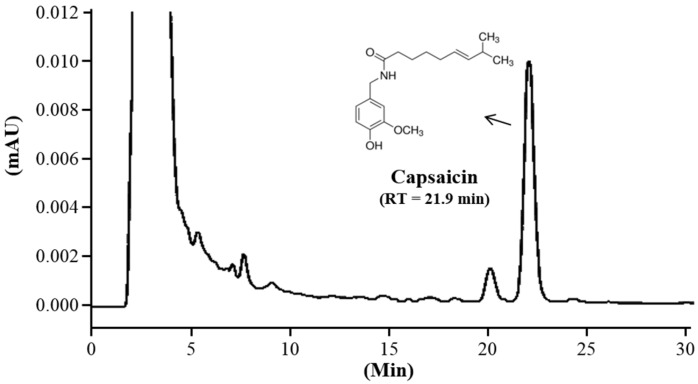
High-performance liquid chromatography (HPLC) chromatogram of capsaicin (CAP) in the supplement sample (mAU: milli absorbance units; Rt: Retention time).

The total capsaicin content of CAP extracts was determined by a high-performance liquid chromatography (HPLC) method. A Hitachi Primaide 1110 HPLC pump system equipped with a Primaide 1430 diode array detector and Primaide 1210 autosampler was used to analyze capsaicin on an Phenomenex Luna C18 column (i.d. 4.6 × 250 mm) at 280 nm. A mixture of 5% methanol in 0.1% phosphoric acid was used as the mobile phase at a flow rate of 1 mL/min and an injection volume of 20 μL. The retention time of CAP was 21.9 min (Figure 1). The content of capsaicin was 85% based on a calibration curve from an absolute standard.

### 3.2. Effect of CAP Supplementation on Body Weight and Organ Weights

**Figure 2:**
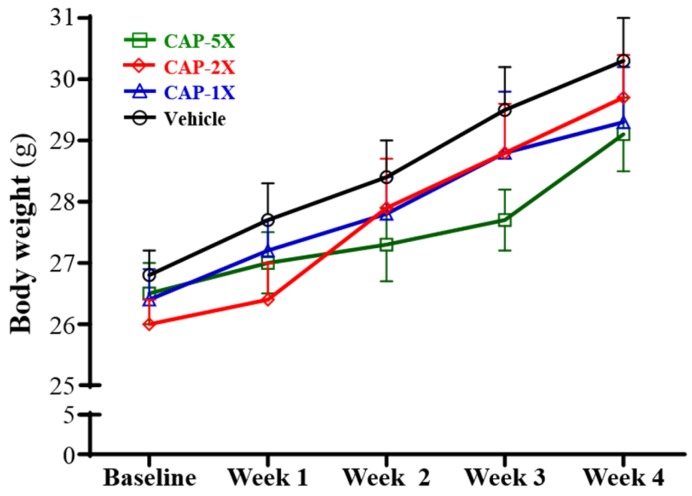
The effect of CAP supplementation on growth curve. Data are mean ± SEM for *n* = 8 mice per group.

The initial and final body weights did not significantly differ among the vehicle, CAP-1X, CAP-2X, and CAP-5X groups (Figure 2). The body weight, food consumption, and body compositions are summarized in Table 1. Significantly, the food and water intake of the CAP-2X group was 7.2% (*p* = 0.0439) and 8.0% (*p* = 0.0226) lower, respectively, as compared with vehicle group. There were no significant differences in food and water intake among the vehicle, CAP-1X and CAP-5X groups.

### 3.3. Effect of CAP on Forelimb Grip Strength

**Figure 3:**
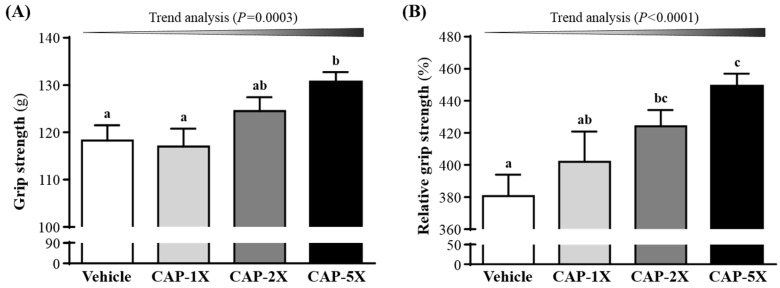
Effect of CAP supplementation on forelimb grip strength. (**A**) Grip strength; (**B**) Relative grip strength. Data are mean ± SEM for *n* = 8 mice in each group. Different letters indicate significant difference at *p* < 0.05 by one-way ANOVA. Low-dose (CAP-1X), medium-dose (CAP-2X) and high-dose (CAP-5X) CAP at 205, 410 and 1025 mg/kg/day, respectively.

The grip strength test is a test designed to assess changes in neuromuscular coordination, muscle strength and overall functional capacity [23]. As shown in Figure 3A, the forelimb grip strength values in the vehicle, CAP-1X, CAP-2X and CAP-5X groups were 118, 117, 125 and 131 g, respectively. The values of the CAP-5X group were significantly 1.10- (*p* = 0.0082) and 1.12-fold (*p* = 0.004) higher than those of the vehicle and CAP-1X groups, respectively. However, there were no significant differences in forelimb grip strength among the vehicle, CAP-1X, and CAP-2X groups. In the trend analysis, absolute forelimb grip strength dose-dependently increased as the CAP dose (*p* = 0.0003) increased. Therefore, grip strength was calibrated by individual body weight to obtain relative grip strength (%) and was still higher with CAP treatment (Figure 3B) than the vehicle group, with significant trend findings (*p* < 0.0001).

### 3.4. Effect of CAP Supplementation on Exhaustive Swimming Test

**Figure 4:**
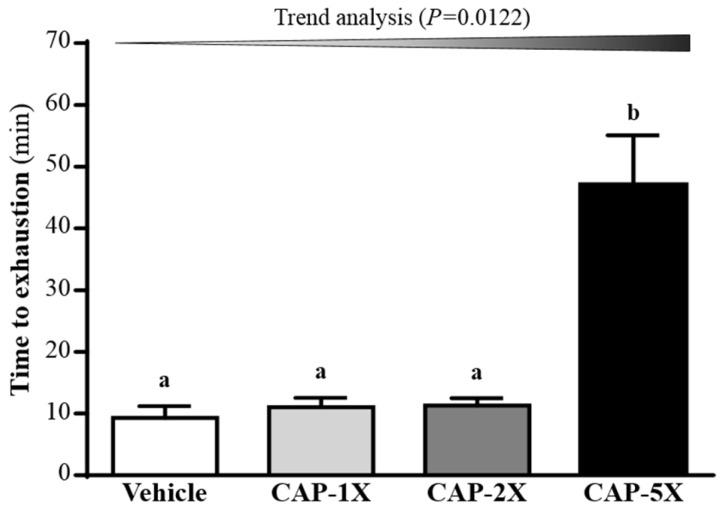
Effect of CAP supplementation on exhaustive swimming test. Data are mean ± SEM for *n* = 8 mice in each group. Different letters indicate significant difference at *p* < 0.05 by one-way ANOVA.

The exercise endurance in mice administered the vehicle, CAP-1X, CAP-2X, and CAP-5X were 9.30, 11.06, 11.28, and 46.99 min, respectively, as shown in Figure 4. The swimming time of the CAP-5X group was significantly higher, 5.06-, 4.25- and 4.17-fold (all *p* < 0.0001) with regard to those of the vehicle, CAP-1X and CAP-2X groups, respectively. In the trend analysis, maximal swimming time was increased dose-dependently with the CAP doses (*p* = 0.0122).

### 3.5. Effect of CAP Supplementation on Exercise Fatigue-Related Indicators after Acute Exercise

**Figure 5:**
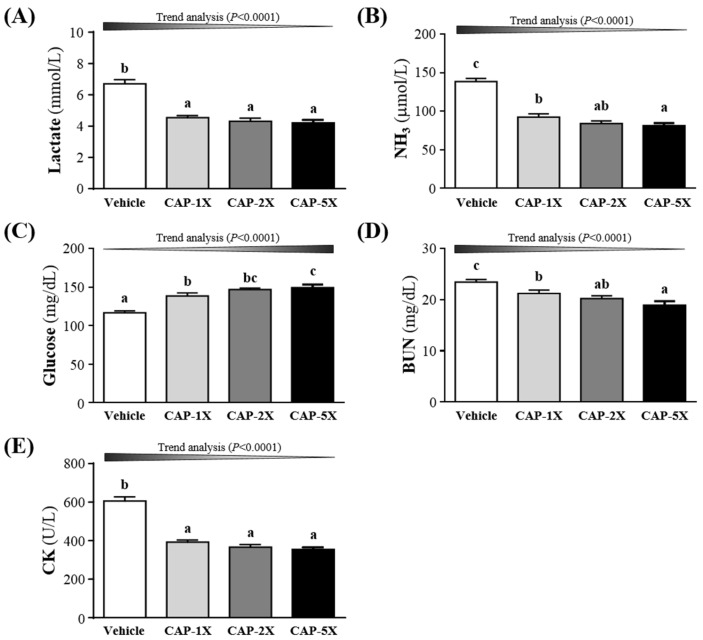
Effect of CAP supplementation on serum (**A**) lactate; (**B**) ammonia; (**C**) glucose; (**D**) blood urea nitrogen (BUN); and (**E**) creatine kinase (CK) levels after acute exercise challenge. Data are mean ± SEM for *n* = 8 mice in each group. Different letters indicate significant difference at *p* < 0.05 by one-way ANOVA.

The status of peripheral fatigue can be evaluated by important biochemical indicators, including lactate, ammonia, glucose, BUN and CK, after exercise [19,20]. Compared to vehicle treatment, serum lactate concentrations were lower for CAP-1X, CAP-2X, and CAP-5X treatment, by 32.3%, 35.8% and 37.3% (all *p* < 0.0001), respectively, as compared to vehicle group (Figure 5A). Ammonia is a ubiquitous metabolic product which has multiple effects on physiological and biochemical systems [24]. As shown in the Figure 5B, serum ammonia levels of CAP-1X, CAP-2X, and CAP-5X groups were significantly lower, by 33.5%, 39.3% and 41.3% (all *p* < 0.0001), respectively, as compared with vehicle group. Figure 5C shows that respective levels of serum glucose were significantly higher, by 19.0%, 26.0% and 28.1% (all *p* < 0.0001), for CAP-1X, CAP-2X, and CAP-5X groups, when compared to the vehicle group. BUN level was lower with CAP treatments than vehicle treatment after exercise (Figure 5D) and significantly differed among CAP treatments. BUN level after exercise decreased by 9.4% (*p* = 0.0194), 13.0% (*p* = 0.0012) and 19.2% (*p* < 0.0001) for CAP-1X, CAP-2X, and CAP-5X groups, respectively, as compared to the vehicle group. CK level significantly differed among CAP treatments (Figure 5E) and was lower, by 35.2%, 39.5% and 41.5% (all *p* < 0.001), for CAP-1X, CAP-2X, and CAP-5X treatments, respectively, in comparison to vehicle treatment. The trend analysis evaluates whether CAP treatment had a significant dose-dependent effect. The statistics results show the increase of dosage-dependence on blood glucose content (*p* < 0.0001) and the decrease of dosage-dependence on serum lactate, ammonia, BUN and CK levels (all *p* < 0.0001).

### 3.6. Effect of CAP Supplementation on Hepatic and Muscular Glycogen Level

**Figure 6:**
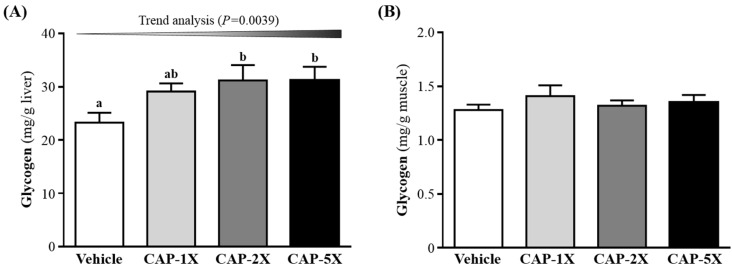
Effect of CAP supplementation on glycogen content in (**A**) liver and (**B**) muscle. Data are mean ± SEM for *n* = 8 mice in each group. Different letters indicate significant difference at *p* < 0.05 by one-way ANOVA.

Glycogen is an important source of energy during exercise, and the increase in glycogen stored in liver is beneficial for enhancing physical endurance [25]. Glycogen content is an integral determining factor in fatigue. In Figure 6A, CAP-2X and CAP-5X groups showed significantly elevated glycogen stores in liver, increased 1.34- (*p* = 0.0191) and 1.34-fold (*p* = 0.0193), respectively, as compared with vehicle group. There were no significant differences between the vehicle and CAP-1X group. In the trend analysis, absolute liver glycogen showed significantly dose-dependent increase (*p* = 0.0039). However, muscle glycogen level did not differ among CAP treatments (Figure 6B).

### 3.7. Effect of CAP Supplementation on Biochemical Analyses at the End of the Experiment

In the present study, we observed beneficial effects of CAP on the grip strength, exhaustive exercise challenge and other physiological effects with 4 weeks of CAP supplementation. We further investigated whether CAP treatments for 4 weeks could cause any negative effects on other biochemical markers of healthy mice. Therefore, we examined the related biochemical parameters to these CAP-treated mice (Table 2).

Levels of biochemical indices, including albumin, TP, TC, and glucose, did not differ among groups (*p* > 0.05, Table 2). Moreover, AST levels were significantly lower, by 16.1% (*p* = 0.0037), 16.1% (*p* = 0.0037), and 19.6% (*p* = 0.0006) for CAP-1X, CAP-2X, and CAP-5X, respectively, when compared to vehicle treatment. In the trend analysis, AST levels were decreased dose-dependently with the CAP doses (*p* = 0.0003). Levels of ALT were significantly lower, by 18.0% (*p* = 0.0051), 30.9% (*p* < 0.0001), and 31.7% (*p* < 0.0001) for CAP-1X, CAP-2X, and CAP-5X, respectively, when compared to vehicle treatment. Serum levels of BUN in the CAP-1X, CAP-2X, and CAP-5X groups were 15.6%, 19.6%, and 25.5% (all *p* < 0.0001), respectively; significantly lower than in the vehicle group. Levels of creatinine in the CAP-1X, CAP-2X, and CAP-5X groups were significantly lower by 13.0% (*p* = 0.0015), 18.6% (*p* < 0.0001), and 29.4% (*p* < 0.0001), respectively, than in the vehicle group. UA levels were significantly lower, by 42.1%, 48.6%, and 55.0% (all *p* < 0.0001) for CAP-1X, CAP-2X, and CAP-5X, respectively, when compared to vehicle treatment. CK levels were significantly lower, by 33.2% (*p* = 0.0003), 51.4% (*p* < 0.0001), and 61.0% (*p* < 0.0001) for CAP-1X, CAP-2X, and CAP-5X, respectively, when compared to vehicle treatment. In the trend analysis, ALT, BUN, creatinine, CK and UA levels decreased dose-dependently with the CAP doses (all *p* < 0.0001). Serum TG levels of CAP-2X, and CAP-5X groups were significantly lower, by 9.4% (*p* = 0.0284) and 12.3% (*p* = 0.0051), respectively, when compared with vehicle group. In the trend analysis, TG level was decreased dose-dependently with the CAP doses (*p* = 0.0014).

### 3.8. Effect of CAP Supplementation on Histological Examinations at the End of the Experiment

**Figure 7:**
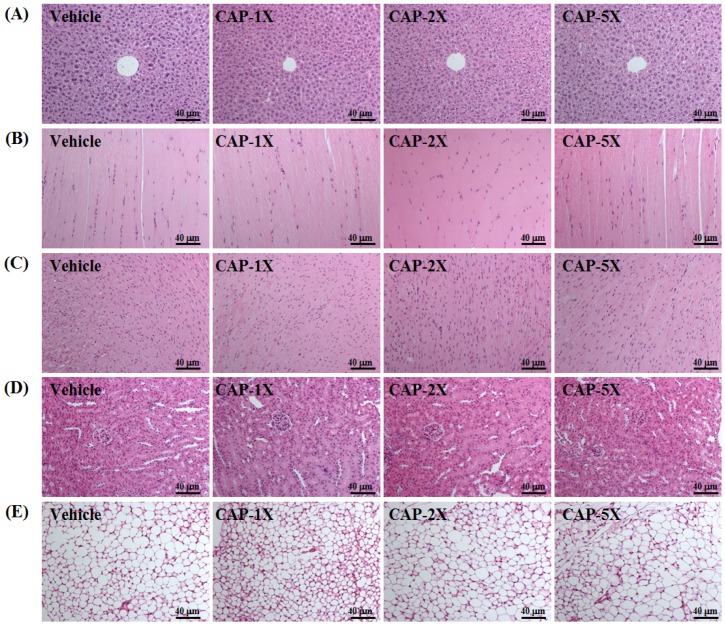
Effect of CAP supplementation on morphology of (**A**) liver; (**B**) skeletal muscle; (**C**) heart; (**D**) kidney; and (**E**) uterine fat pad in mice. Specimens were photographed by light microscopy. (H&E stain, magnification: ×200; Scale bar, 40 μm).

On morphological observation, the arrangement of sinusoid and hepatic cords in liver showed no changes with CAP treatment (Figure 7). Hypertrophy and hyperplasia were not observed in heart cardiomyocytes or skeletal muscles. The structure of renal tubules and glomerulus did not differ among treatments. In addition, the morphology of adipose tissue and fat cell size did not show differences between groups.

## 4. Discussion

Previous studies have reported that optimized dose of CAP may significantly affect fat oxidation and energy expenditure [12,26]. Thus, we found the CAP-2X groups showed a significant decrease in food consumption, but not in other CAP groups, which was consistent with previous study. Organ weights of experimental animals could provide information about the health status of test mice and possible effects to the test article. The weight differences of the liver, muscle, kidney, heart, uterine fat pads (UFP) and brown adipose tissue (BAT) were not observed among the four groups. The relative liver, muscle, kidney, UFP and BAT weight (%) also did not show significant differences between groups. The no-observed-adverse-effect level (NOAEL) of CAP could provide optimized dosages for its physiological benefits without health risk. The results suggested that the supplementation with CAP treatments should be safe for all test animals.

Forelimb grip strength is a routine physical examination test. Our previous study had found that muscle strength was positively correlated with forelimb grip strength [19]. In this study, we found the grip of CAP-5X group was greater than other groups. Therefore, the results indicated that long-term CAP supplementation could benefit grip strength when no training protocol is implemented. Our previous reports have shown that long-term phytochemical supplementation, such as resveratrol and curcumin, also improves the grip strength of untrained animals [19,20]. CAP also demonstrated the improvement of mechanical performance and bioenergetic efficiency in contracting mouse skeletal muscle [27], which is consistent with our grip results. Our experimental results (Figure 4) indicated that physical fatigue could be ameliorated according to swimming time extension with 4 weeks CAP administration. TRPV1 activation by dietary capsaicin supplementation had revealed one of its roles in skeletal muscle function [14]. The TRPV1 activation promoted mitochondrial biogenesis and ATP production through PGC-1α regulation in skeletal muscle. A previous study has shown that TRPV1, activated by CAP administration, could improve endurance capacity and energy metabolism in mice [13]. CAP was able to significantly extend the endurance time to exhaustion, so it had anti-fatigue activity due to endurance enhancement. Swimming test to exhaustion is an experimental exercise model to evaluate physical fatigue. It is one of the most commonly used animal models that works well for evaluating the physical endurance capacity [18,28]. In short-term intensive exercise, blood lactate is the glycolysis product of carbohydrate under an anaerobic condition and glycolysis is the main energy source [29]. The increased lactate levels further reduce pH, which could induce various biochemical and physiological side effects, including glycolysis, as well as phosphofructokinase and calcium ion release, through muscular contraction [30]. Therefore, lactate metabolism is beneficial to relieve fatigue. We found that CAP supplementation prolonged the period to exhaustion and suppressed the increase in the blood lactate level during swimming (Figure 5A). Another study also demonstrated that CAP administration could also modulate serum lactic acid levels after 30 min of swimming via stimulation of their vanilloid receptors [31].

High-intensive and prolonged exercise significantly enhances the blood ammonia concentration, a metabolite with toxic effects on the central and peripheral fatigue [32]. During exercise, ammonia is produced and accumulates in skeletal muscle when AMP is deaminated to IMP by AMP deaminase (AMPD) during resynthesis of ATP. Ammonia is very toxic and has deleterious influences on the physiology, including activation of phosphofructokinase (PFK), which is the rate-limiting enzyme in glycolysis, and inhibition of pyruvate oxidation to acetyl-CoA [33]. Our previous reports have also shown that long-term phytochemical supplementation, such as resveratrol and curcumin, could reduce the concentration of ammonia after exercise [19,20]. Higher concentrations of ammonia content could bring negative effects on the productivity pathway. Thus, it is suggested that when CAP supplementation for 4 weeks, the ammonia level could be significantly modulated after the exercise test (Figure 5B).

Glucose, a breakdown product of tissue glycogen, is released as a circulating substrate for energy utilization after intense exercise [34]. During exercise, energy demand expedite muscular glucose utilization and, consequently, increases blood glucose disposal [35]. Blood glucose is an important fuel for increased ATP production within contracting skeletal muscle during exercise [36]. Previous reports showed that the plasma glucose of CAP-treated rats at a dose of 15 mg/kg was significantly higher than the non-CAP-treated control group after immediate exhaustion [37]. The other studies also demonstrated that CAP increased glucose uptake directly by activating AMP-activated protein kinase (AMPK) [38], improved oxidative metabolism in exercising muscle, and promoted muscular mass gain with chronic administration [39]. Therefore, continuous 4 weeks CAP supplementation could increase energy utilization and improve exercise performance in current results (Figure 4).

Serum BUN (blood urea nitrogen), creatinine, and urine output were closely monitored to measure renal function. Many factors other than renal disease can cause BUN alteration [40]. Urea is formed by the liver and carried by the blood to the kidneys, and urea is an important index correlation with protein breakdown, dehydration, stress and fatigue [40]. CAP is also reported to mitigate the cisplatin-induced renal dysfunction, by assessing the levels of serum creatinine and BUN, through induction of HO-1 as well as inhibition oxidative stress and inflammation [41].

The appearance of creatine kinase (CK) in blood has been generally considered to be an indirect marker of muscle damage, particularly for diagnosis of medical conditions such as myocardial infarction, muscular dystrophy, and cerebral diseases [42]. Therefore, serum CK activity has been considered to be useful as a marker in exercise physiology and sports medicine for the detection of muscle injury and overwork [43]. Thus, CAP supplementation should ameliorate skeletal muscle injury induced by acute exercise challenge.

Energy storage and supply is another important factor related to exercise performance. Glycogen is the predominant source of glycolysis for ATP production. High pre-exercise muscle and liver glycogen concentrations are believed to be essential for exercise performance [44]. In a previous study, the oral administration of CAP (15 mg/kg) could substantially improve the endurance performance by stimulating lipolysis and sparing tissue glycogen [45]. The non-esterified fatty acids could be utilized prior to glycogen due to CAP-increased adrenal catecholamine levels [37,45]. Administration of CAP or its analog, capsiate, promotes hepatic insulin sensitivity and increases the glycogen storage via pAkt, PEPCK and pAMPK signaling pathways [46]. CAP increases liver insulin sensitivity, but not muscular insulin sensitivity responsiveness, thereby creating a metabolism milieu that favors in vivo insulin sensitivity with respect to glucose uptake or production, glycogenesis and lipogenesis [47]. Exercise expedites the gluconeogenesis and glucose utilization to meet the body’s energy demands. In the acute phase of exercise, liver glycogen may break down to glucose as an alternative energy source, and then blood flow carries nutrients, including glucose, to working muscles supporting exercise [21]. Our data also showed similar result; breakdown of glycogen resulted in elevated blood glucose for sufficient energy during exercise. This may help to explain that the elevated blood glucose levels in the CAP-treated groups after a 15-min exercise challenge were all significantly higher as compared to the vehicle control group (Figure 5C). We found that CAP supplementation could increase glycogen content in liver. These results suggest that the glycogen deposit could become an available energy source for the following phases of exercise, which increase endurance capacity and delays onset of fatigue.

Liver function could be evaluated by measuring plasma levels of aminotransferase such as AST and ALT. In a previous study, CAP treatment showed significant improvement in the anti-oxidation and anti-apoptosis against carbon tetrachloride-induced hepatotoxicity in rats [48]. In the current study, it was also demonstrated CAP supplementation could be beneficial to the liver function. The kidney-related indexes including BUN, creatinine, and UA were all improved by CAP supplementation. During renal ischemia-reperfusion (I/R) injury, the burst of reactive oxygen species (ROS), mainly produced by xanthine oxidase, can trigger inflammation and tubular cell injury and subsequent generation of UA [49]. Therefore, CAP may have potential for renal protection due to its antioxidant activity.

Blood CK activity is a marker of exercise-induced skeletal muscle damage [42]. The CAP showed improvement of cardiac function recovery and CK amelioration in heart reperfusion injury, possibly related to stimulation of calcitonin gene-related peptide (CGRP) release [50]. Therefore, CAP supplementation should mitigate the muscular injury induced by acute exercise or ischemia. A previous study also showed that dietary capsaicin significantly decreased triglyceride levels in the plasma and/or liver, as well as the expression of inflammatory adipocytokine genes (e.g., monocyte chemoattractant protein-1 and interleukin-6) and macrophage infiltration [11]. The capsaicin-induced TRPV1 activation promoted lipolysis of visceral fat, and reduced triglyceride level via upregulation of hepatic uncoupling protein 2 (UCP2) [51].

Histological data related to the pathological effects of CAP is quite limited, especially with respect to the indicated bioactivity doses. We found no changes in arrangement of sinusoid and hepatic cords with CAP treatment in mice and no hypertrophy or hyperplasia response in heart cardiomyocytes and rhabdomyocytes of gastrocnemius muscles. We found no significant difference in structure of renal tubules and glomerulus with CAP treatment and no significant alteration in the alveolar, bronchial and interstitial space. The morphology of adipose tissue morphology and fat cell size were not shown to be different between groups. Histological examination of organs showed no apparent damage in any mice.

## 5. Conclusions

In this study, we found that CAP supplementation could improve physical activities, including grip strength and endurance performance, by increasing liver glycogen content. In addition, exercise-induced fatigue-related parameters, including lactate, ammonia, glucose, BUN and CK, were positively modulated by CAP supplementation in a dosage-dependent manner (trend analysis, *p* < 0.0001). We suggest that CAP could be a potential ergogenic phytocompound for supplementation, with fatigue mitigation and exercise performance enhancement.

## Figures and Tables

**Table 1:** General characteristics of the experimental groups with capsaicin supplementation.

Characteristic	Vehicle	CAP-1X	CAP-2X	CAP-5X	Trend Analysis
Initial BW (g)	26.8 ± 0.4	26.4 ± 0.5	26.0 ± 0.4	26.5 ± 0.5	0.1161
Final BW (g)	30.3 ± 0.7	29.3 ± 0.9	29.7 ± 0.7	29.1 ± 0.6	0.3282
Food intake (g/day)	5.57 ± 0.02 ^b^	5.52 ± 0.26 ^a,b^	5.17± 0.00 ^a^	5.34 ± 0.03 ^a,b^	0.6056
Water intake (mL/day)	6.37± 0.08 ^b^	6.14 ± 0.22 ^a,b^	5.86 ± 0.03 ^a^	6.14 ± 0.20 ^a,b^	0.0875
Liver (g)	1.41 ± 0.04	1.37 ± 0.04	1.37 ± 0.04	1.46 ± 0.02	0.2536
Muscle (g)	0.30 ± 0.01	0.30 ± 0.00	0.30 ± 0.01	0.30 ± 0.00	0.6938
Kidney (g)	0.38 ± 0.01	0.38 ± 0.00	0.38 ± 0.01	0.38 ± 0.01	0.9420
Heart (g)	0.18 ± 0.01	0.16 ± 0.01	0.15 ± 0.00	0.16 ± 0.00	0.7942
UFP (g)	0.20 ± 0.01	0.19 ± 0.02	0.20 ± 0.02	0.20 ± 0.01	0.7942
BAT (g)	0.08 ± 0.003	0.09 ± 0.002	0.08 ± 0.004	0.09 ± 0.003	0.9519
Relative liver weight (%)	4.73 ± 0.18	4.92 ± 0.10	4.73 ± 0.18	5.03 ± 0.14	0.3336
Relative muscle weight (%)	1.00 ± 0.01	1.02 ± 0.02	1.03 ± 0.03	1.04 ± 0.02	0.3257
Relative kidney weight (%)	1.28 ± 0.05	1.28 ± 0.03	1.31 ± 0.03	1.30 ± 0.05	0.9771
Relative heart weight (%)	0.59 ± 0.03 ^b^	0.54 ± 0.03 ^a,b^	0.51 ± 0.02 ^a^	0.56 ± 0.02 ^a,b^	0.6981
Relative UFP weight (%)	0.68 ± 0.03	0.66 ± 0.08	0.69 ± 0.08	0.67 ± 0.05	0.9121
Relative BAT weight (%)	0.28 ± 0.01	0.30 ± 0.01	0.29 ± 0.02	0.30 ± 0.01	0.5119

Data are the mean ± SEM for *n* = 8 mice in each group. Values in the same row with different superscript letters (a, b) differ significantly, *p* < 0.05, by one-way ANOVA; Muscle mass includes both gastrocnemius and soleus muscles in the back part of the lower legs. BW: Body weight; UFP: Uterine fat pads; BAT: Brown adipose tissue. Low-dose (CAP-1X), medium-dose (CAP-2X) and high-dose (CAP-5X) CAP at 250, 410 and 1025 mg/kg/day, respectively.

**Table 2:** Biochemical analysis at the end of treatment.

Parameter	Vehicle	CAP-1X	CAP-2X	CAP-5X	Trend Analysis
AST (U/L)	95 ± 5 ^b^	80 ± 2 ^a^	80 ± 3 ^a^	77 ± 3 ^a^	0.0003
ALT (U/L)	45 ± 3 ^d^	37 ± 2 ^c^	31 ±1 ^a,b^	30 ± 2 ^a^	<0.0001
Albumin (g/dL)	3.26 ± 0.04	3.28 ± 0.04	3.26 ± 0.04	3.28 ± 0.03	0.8838
TP (g/dL)	5.04 ± 0.06	4.98 ± 0.07	5.04 ± 0.05	5.05 ± 0.02	0.5837
BUN (mg/dL)	22.3 ± 0.6 ^c^	18.8 ± 0.3 ^b^	17.9 ± 0.2 ^b^	16.6 ± 0.5 ^a^	<0.0001
Creatinine (mg/dL)	0.29 ± 0.01 ^c^	0.25 ± 0.01 ^b^	0.24 ± 0.01 ^b^	0.20 ± 0.01 ^a^	<0.0001
UA (mg/dL)	1.75 ± 0.11 ^b^	1.01 ± 0.09 ^a^	0.90 ± 0.07 ^a^	0.79 ± 0.04 ^a^	<0.0001
CK (U/L)	259 ± 24 ^d^	173 ± 12 ^c^	126 ± 10 ^a,b^	101 ± 7 ^a^	<0.0001
TC (mg/dL)	111 ± 3	103 ± 4	104 ± 5	102 ± 5	0.1701
TG (mg/dL)	152 ± 5 ^b^	147 ± 4 ^b,c^	138 ± 6 ^a,c^	134 ± 3 ^a^	0.0014
Glucose (mg/dL)	154 ± 3	153 ± 4	158 ± 2	158 ± 3	0.5448

Data are the mean ± SEM for *n* = 8 mice in each group. Values in the same row with different superscript letters (a, b, c) differ significantly, *p* < 0.05, by one-way ANOVA; Muscle mass includes both gastrocnemius and soleus muscles in the back part of the lower legs. AST: Aspartate aminotransferase; ALT: Alanine aminotransferase; TP: Total protein; BUN: Blood urea nitrogen; CK: Creatine kinase; UA: Uric acid; TC: Total cholesterol; TG: Triacylglycerol.
